# Sublobar resection versus lobectomy in Surgical Treatment of Elderly Patients with early-stage non-small cell lung cancer (STEPS): study protocol for a randomized controlled trial

**DOI:** 10.1186/s13063-016-1312-6

**Published:** 2016-04-07

**Authors:** Fan Yang, Xizhao Sui, Xiuyuan Chen, Lixue Zhang, Xun Wang, Shaodong Wang, Jun Wang

**Affiliations:** Department of Thoracic Surgery, Center for Mini-invasive Thoracic Surgery, People’s Hospital, Peking University, #11 Xizhimen South Avenue, Beijing, 100044 China

**Keywords:** Elderly, Lung cancer, Sublobar resection, Lobectomy, Survival, Pulmonary function, Geriatric assessment

## Abstract

**Background:**

The appropriateness of lobectomy for all elderly patients is controversial. Meanwhile, sublobar resection is associated with reduced operative risk, better preservation of pulmonary function, and a better quality of life, constituting a potential alternative to standard lobectomy for elderly patients with early-stage non-small cell lung cancer (NSCLC). To date, no randomized trial comparing sublobar resection and lobectomy focusing on elderly patients has been reported. We hypothesized that for patients at least 70 years old with clinical stage T1N0M0 NSCLC, sublobar resection is non-inferior to lobectomy for 3-year disease-free survival (DFS).

**Methods/design:**

This is a prospective, randomized, controlled multicenter non-inferiority trial with two study arms: sublobar resection and lobectomy groups. Comprehensive geriatric assessments will be acquired for each patient. A total of 339 subjects will be enrolled on the basis of power calculations, and participants followed up every 6 months post-operation for 3 years. In case of relapse, survival follow-up will be continued until 5 years or death. Pulmonary function testing will be performed at 6, 12, and 36 months post-operation. The primary outcome is 3-year DFS; secondary endpoints include peri-operative complications and mortality, hospitalization time, post-operative ventilator time, overall survival, 3-year recurrence rates, post-operative pulmonary function, quality of life, geriatric assessment data, and 4-year mortality index.

**Discussion:**

The present study is the only prospective, multicenter, randomized controlled trial comparing sublobar resection and lobectomy for elderly patients. The therapeutic outcomes of sublobar resection will be evaluated in comparison with lobectomy for elderly patients (≥70 years) with early-stage NSCLC.

**Trial registration number:**

NCT02360761: 01/24/2015 (ClinicalTrials.gov)

## Background

Lung cancer is the most commonly diagnosed malignancy and the most common cause of cancer death worldwide, and non-small cell lung cancer (NSCLC) accounts for 80–85 % of those deaths. According to the International Agency for Research on Cancer, an estimated 1.8 million new lung cancer cases and 1.6 million lung cancer-related deaths occurred in 2012 [1].

Lung cancer incidence increases with age. According to the Surveillance, Epidemiology, and End Results (SEER) database, age-adjusted incidence of NSCLC in populations at least 65 years old was about 20 times higher compared with that of younger groups between 2004 and 2008 [2]. As a result of population aging, the proportion of elderly patients is also rapidly increasing. The median age of NSCLC diagnosis rose from 64 years (1974–1978) to 70 in the late 20th century (1999–2003). Forty-seven percent of NSCLC were diagnosed in patients at least 70 years, and 14 % were above age 80 [3]. In 2005–2009, about 71 % of NSCLC cases were newly diagnosed in patients over 70 years, and more than one third were at least 80 years [4]. Although elderly patients have earlier-stage lung cancer at diagnosis compared with younger patients [5, 6], they themselves are somehow under-represented in studies of treatment for cancer [7].

Surgery is the primary option for treating early-stage NSCLC [8, 9]. The Lung Cancer Study Group (LCSG) demonstrated a threefold increase of local recurrence and a tendency toward decreased survival among patients who underwent sublobar resection, and supported lobectomy as the standard of care for stage I NSCLC [10]. However, the appropriateness of lobectomy for all elderly patients is questionable, given that declined function of organs is an independent determinant of post-operative mortality and morbidity, and shorter life expectancy limits the benefit from curative-intent resection [11, 12].

First, lobectomy has been less employed in many elderly patients facing an opportunity for surgical treatment. Although advanced age by itself is not an absolute contraindication to major thoracic surgery [13] and disease-specific survival of elderly patients can be unrelated to age [14–17], many fewer elderly patients with stage I/II NSCLC receive standard lobectomy than the young counterparts. In the US, Nugent et al. reported a resection rate of 6 % in patients no younger than 80 years, although one third of them presented with early-stage disease (stage I or II); meanwhile, one third of patients below 45 underwent thoracic operation, and only 6 % had stage I or II disease [18]. Similarly, in Europe, Damhuis and Schutte found resection rates of 26 % in lung cancer patients younger than 70 years and merely 14 % in those older than 70 years [19].

Second, advanced age is associated with a substantial increase in morbidity and mortality from lobectomy [12]. It was reported that, for patients at least 80 years old, major complication rate is 30 % and that overall mortality rate (in hospital or within 30 days) was 16 % after lobectomy [20]. A retrospective study from the Mayo Clinic reported morbidity and mortality rates of 48.0 % and 6.3 %, respectively, for patients at least 80 years old who underwent lobectomy for lung cancer [21]. Similar conclusions were reached in a population-based analysis, where peri-operative complication rate exceeded 50 % in elderly patients [22].

Third, for elderly patients with early-stage NSCLC, competing risks of dying from co-morbidities may exceed the risk of cancer-related deaths. Evidence from the SEER database indicated that the 5-year probability of dying from cardiovascular diseases is significantly higher than that of dying from cancer in patients 70 to 79 years old who survived for 7 years and those at least 80 years old who survived for 5 years after lobectomy [23]. Limited life expectancy of the elderly restricts the benefit from radical resection.

Lobectomy is not the only option for elderly patients with early-stage NSCLC. Several retrospective studies supported sublobar resection, including segmentectomy and wedge resection, as a sound alternative for the elderly. A retrospective study from the SEER database analyzing 14,555 patients with NSCLC stage I/II suggested that lobectomy conveys benefits only patients younger than 71 years [24]. Similarly, at Pittsburgh Medical Center, Kilic et al. indicated that segmentectomy is associated with reduced peri-operative complications (29.5 % versus 50 %) in patients over 70 years with stage I NSCLC but that local recurrence and overall survival rates are not significantly different [25]. A retrospective study from Japan found that 5-year survival after sublobar resection is significantly inferior to that after standard lobectomy (64.0 % and 90.9 %, respectively, *P* < 0.0001) in the younger group (median age of 68 versus 64); however, in the elderly (median age of 78 versus 77), no substantial difference was observed (67.6 % and 74.3 %, *P* = 0.92) [26]. These data demonstrated that sublobar resection might achieve equivalent benefits compared with lobectomy for the elderly and that the gap in long-term outcome between sublobar resection and lobectomy might shrink as patient age increases. Additionally, sublobar resection may play a better role in preserving the lung function [27, 28], which is important for patients with small lung cancer, conferring long survival, considering the possibility of further lung resections to treat second primary lung tumors.

In summary, sublobar resection may be an alternative to standard lobectomy for elderly patients with early-stage NSCLC, resulting in less operative risk, better preservation of pulmonary function, and improved quality of life (QoL), without compromising survival. However, trials dedicated to surgical assessments are scarce in elderly patients, who comprise a large percentage of all patients.

This randomized trial is, to the best of our knowledge, the first designed to compare sublobar resection and lobectomy for elderly patients in order to address the following open questions: whether in patients at least 70 years old with clinical stage T1N0M0 NSCLC (1) sublobar resection can achieve similar disease-free survival (DFS) compared with lobectomy, (2) sublobar resection can reduce post-operative mortality and morbidity, and (3) sublobar resection can yield better pulmonary function and QoL.

## Methods

### Trial design

The Surgical Treatment of Elderly Patients with early-stage non-small cell lung cancer (STEPS) study was designed as a prospective, randomized, controlled, multicenter non-inferiority trial with two parallel study arms. The hypothesis was that for patients 70 or over with clinical stage T1M0M0 NSCLC, sublobar resection could achieve non-inferior 3-year DFS.

### Eligibility criteria and patient consent

All consecutive patients referred to the surgical units will be assessed to determine eligibility, by one dedicated team member, according to the inclusion and exclusion criteria described below. Each patient will be introduced to the trial by a research group member and receive an explanation of the study protocol. Specific informed consent regarding participation in the trial, randomization, and explanation of the surgery will be obtained before enrolment.

The study will be carried out in accordance with the Helsinki Declaration. It was approved by the Ethics Committee of Peking University Institutional Review Board on 30 October 2014 prior to registration (reference number IRB00001052-13053).

#### Inclusion criteria

- Age of 70 years or older
- Pre-operation criteria (contrast-enhanced computed tomography (CT) scan)
  - ▪ Suspected peripheral NSCLC.
  - ▪ Clinical stage IA (i.e., T1 N0 M0: tumor diameter of not more than 3 cm, surrounded by visceral pleura, short axis of lymph node of less than 1 cm or negative lymph nodes on positron emission tomography scan)
  - ▪ Maximum diameter of consolidation to maximum tumor diameter ratio (consolidation/tumor ratio, consolidation/tumor ratio) no less than 0.5 in sub-solid lesions.
  - ▪ Eligibility for sublobar resection with sufficient margin.
- Intra-operative criteria
  - ▪ Histologically confirmed invasive NSCLC (i.e., NSCLC other than pre-invasive adenocarcinomas defined by the International Association for the Study of Lung Cancer: adenocarcinoma *in situ* and minimal invasive adenocarcinoma) [29].
  - ▪ Pathological exclusion of suspected lymph node involvement.
  - ▪ Feasibility of sublobar resection in terms of surgical margin requirement.
- General criteria
  - ▪ Signed written informed consent by the patient or his or her entrusted party.
  - ▪ Completed 4-year mortality index [30] and comprehensive geriatric assessment (CGA) if necessary.
  - ▪ Physiology which can tolerate lobectomy (percentage of forced expiratory volume in 1 second (FEV1%) of at least 50 % and diffusing capacity of the lungs for carbon monoxide (DLCO) of at least 50 % in pulmonary function test).

#### Exclusion criteria

- Inability to comply with the study procedure.
- Thoracic surgery history, except for diagnostic thoracoscopy.
- Malignant tumor history within the past 5 years, except for the following conditions: cured skin basal cell carcinoma, superficial bladder carcinoma, and uterine cervix cancer *in situ*.
- Any active systemic diseases, including uncontrolled hypertension; unstable angina pectoris; newly onset of angina pectoris within the past 3 months; congestive heart failure (class II or more of New York Heart Association); myocardial infarction within the last 6 months; or severe disease in need of medication, such as arrhythmia, liver, renal, or metabolic diseases.
- Uncontrollable infections.
- Co-existing small-cell lung cancer.
- Psychiatric diseases.
- Other circumstances deemed inappropriate for enrollment by the researcher.

### Comprehensive geriatric assessments

For patients 70 or older with suspected clinical stage I NSCLC, baseline data—including Vulnerable Elders Survey (VES-13) scale, 4-year survival index, and QoL questionnaires: Functional Assessment of Cancer Therapy-Lung Cancer (FACT-L) and Lung Cancer Symptom Scale (LCSS)—should first be acquired. It is not necessary to conduct further assessments if the patient has a VES-13 score of less than 3; otherwise, the patient will be regarded as ‘high-risk population’, and a further evaluation named ‘pre-operative assessment of cancer elderly (PACE)’ will be mandatory [31, 32]. PACE provides a description of the general health condition of elderly cancer patients prior to receiving surgery comprising assessment disability, instrumental function, depression and cognitive status (activities of daily living, instrumental activities of daily living, Geriatric Depression Scale, and Mini-Mental State Examination score), and an additional important pre-treatment component concerning the level of fatigue (brief fatigue inventory), performance status, concurrent co-morbidities, and anaesthesiologist evaluation of operative risk (American Society of Anesthesiologists, or ASA). Following baseline data acquisition, the 4-year mortality index is required as one of the stratification factors.

### Definition of endpoints and outcome measures

#### Primary endpoint

- DFS at 3 years, defined as the time interval from randomization to the earliest onset of any of the following events at 3 years: tumor recurrence, metastasis, or death caused by any reason.

#### Secondary endpoints

- Peri-operative measurements
  - ▪ Peri-operative complications and mortality. A peri-operative complication is defined as one occurring after surgery and before discharge or within 30 days post-operatively. Death cases during this period should be recorded as peri-operative deaths.
  - ▪ Hospitalization time, defined as the time interval from the day of operation to discharge.
- Chest tube duration, defined as the time interval from the day of operation to that of chest tube removal.
- Overall survival at 3 years, defined as the time interval from randomization to death from any cause at 3 years.
- 3-year local recurrence and metastasis rates, defined as the rates of loco-regional recurrence and metastasis, respectively, in 3 years from the day of randomization.
- 6-, 12-, and 36-month post-operative pulmonary function, defined as the pulmonary function 6, 12, and 36 months post-randomization.
- Proportion of video-assisted thoracoscopic surgery procedure, defined as the proportion of video-assisted thoracoscopic surgery in each group.
- QoL, defined as the QoL questionnaire survey.
- Prospective CGA and 4-year mortality index, defined as the CGA by VES-13 scale and PACE (if necessary) and the prognostic evaluation of the elderly by 4-year mortality index, respectively.

#### Exploratory analysis

- To investigate the relationship between radiologic/pathologic parameters of primary NSCLC and prognosis. The consolidation component on CT is defined as an area of increased opacification that completely obscures the underlying vascular markings. Ground-glass opacity is defined as an area of a slight, homogenous increase in density that does not obscure the underlying vascular markings.
- To accomplish the cost/benefit analysis of sublobar resection.
- To investigate the outcomes from participants who underwent video-assisted thoracoscopic or open procedures.

### Randomization

The patients who meet the general and pre-operative criteria will undergo surgery, and intra-operative criteria should be evaluated to exclude pre-invasive adenocarcinoma, lymph node involvement, and inadequate surgical margin before randomization. In this procedure, all pre- and intra-operative suspected lymph nodes are biopsied by frozen section sampling before tumor resection to determine whether the patient can be included and randomly assigned in this trial.

The patients meeting the intra-operative criteria will be included and randomly assigned during surgery. The central randomization system will be used with the dynamic minimization random program software; a random number will be assigned to each patient, and the medical center for the surgery will be informed. The patients will be randomly assigned into sublobar resection or lobectomy group by a central computer and further sub-grouped according to age (≤80 or >80 years), pathological type (adenocarcinoma and other types), tumor size (diameter of ≤2 and >2 cm), and 4-year survival index. Once a random number is assigned to a patient, this specific number cannot be re-assigned another one, even if the patient fails to complete the entire study.

### Intervention

#### Surgical approach

Sublobar resection includes two surgical procedures: wedge resection and segmentectomy. For nodules of not more than 2 cm, the surgeon can choose freely between both operation types as long as surgical margin requirements are met. With a maximum tumor diameter of more than 2 cm, segmentectomy should be preferred. Segmental artery and bronchus should be divided individually to be classified as segmentectomy. The surgical margin should be measured on the resected sample and is required to be no less than the lesion diameter for tumors of not more than 2 cm and no less than 2 cm for 2- to 3-cm lesions. Frozen section of the surgical margin is not mandatory. Sublobar resection should be conversed to lobectomy when the frozen section indicates lymphatic metastasis or marginal involvement. Systematic mediastinal lymph node dissection is mandatory if no sampling was carried out prior to randomization. A minimum of three N2 lymph stations are required; nodal levels 2, 4, and 7 should be examined on the right side, and levels 5, 6, and 7 on the left side, along with at least three N1 lymph stations.

The standard of lymph node dissection/sampling applies to the lobectomy arm, as does the surgical margin requirement. When the tumor locates close to a fused fissure, the adjacent lung tissue should be extendedly resected. The distance from the dissection margin to tumor edge must be evaluated in the same manner as with sublobar resections.

#### Adjuvant therapy

For patients with nodal disease, adjuvant chemotherapy is suggested. However, given the advanced age of the study group, the decision should be made by a multi-disciplinary team. The recommended initiation time should be no earlier than 30 days post-operatively, so that surgical complications are not mistaken for adverse events of adjuvant therapy. For patients with incomplete resection, re-resection or radiation is recommended.

### Follow-up

#### Post-operative follow-up

Chest CT scan and QoL evaluation will be performed at follow-up visits every 6 months after operation for 3 years. In case of relapse, survival follow-up by telephone will be proceeded. The pulmonary function will be examined at follow-up visits in 6, 12, and 36 months after operation.

#### Survival follow-up

Telephone follow-up will be used to contact patients every 3 months if recurrence occurred until 5 years or death, to acquire subsequent medical management and death data. The following information should be obtained at each interview: survival state; death date and cause; disease situation, including recurrence date; detailed information about therapeutic strategy, including the first- and second-line therapies, and any other subsequent operations.

### Adverse event reporting

A data and safety monitoring committee will monitor patient safety, and all ongoing serious adverse events will be closely followed until stabilization. The complications occurring after operation until discharge, or within 30 days post-operation in discharged patients, are considered post-operative complications; death during the same period is defined as peri-operative death. Post-operative complications are described according to the Common Terminology Criteria for Adverse Events 4.1 published (CTCAE version 4.1) by National Cancer Institute of the US, reported, classified, and recorded into case report form (CRF).

### Sample size

Sample size was calculated by the PASS (Power Analysis and Sample Size) software, based on 3-year DFS of elderly patients. There were no exact published data about the DFS rate at 3 years of elderly patients with stage I NSCLC [33, 34], and we estimated the DFS rate at 3 years for lobectomy (control group) to be approximately 75 %. DFS rate at 3 years of 60 % for sublobar resection is considered unacceptable. Under our current assumption, the 15 % of non-inferiority margin on DFS at 3 years corresponds to a hazard ratio of 1.78. The sample-size calculation was based on log rank test. The calculated sample size was 339 patients, and event number of 96 was based on an estimated post-operative 3-year DFS rate of 75 %, 10 % non-compliance, 3-year open enrolment period, 3-year follow-up period, and one-sided type I error rate of 0.025 with 90 % statistical power.

### Recruitment and timeline

This study is planned to start in 10 medical centers across mainland China, and 339 elderly patients with NSCLC at early stage will be recruited. Informed consent should be signed by each patient. Enrollment was activated in December 2015 and is expected to last 3 years. All patients will be included in post-operative and survival follow-ups for 3 years or until death. The actual timeline might be slightly different.

### Database management

A data management plan is drafted in detail before analysis implementation, according to research scheme characteristics and CRF collection. The draft plan includes data quality audit, electronic database establishment, and acquisition method for complete and expected data. Data quality will be checked after primary data input into the database and verification of completely consistent entries. Data consistency and logistics should be confirmed by a combination of artificial audit and computer program. Any doubts raised during this period should be resolved by researchers, and the corresponding update and modification will be carried out by database specialists. The assessment program should be repeated multiple times to avoid doubts, and all modified data and updates recorded and archived. The CRF table will be used to record all data with blue- or black-ink ballpoint pen in clear handwriting. Correction fluid or tape is prohibited during the whole process. Researchers have to ensure CRF table authenticity.

### Statistical methods

Statistical analyses will be performed by using SPSS (SPSS Inc., Chicago, IL, USA). Baseline data of the two groups will be compared by Fisher’s exact and Wilcoxon rank-sum tests as well as two samples *t* test. Primary and secondary key analysis will be carried out according to the intention-to-treat principle, and hazard ratio and 95 % confidence interval values determined. The likelihood ratio will be calculated by using Cox proportional hazards regression analysis as bilateral *P* value. Peri-operative data, including measurements and procedure types, will be analyzed at recruitment completion. Survival data will be analyzed only at trial end.

### Termination criteria

The patient has the right to terminate the research and evaluation at any time point. Exit criteria include voluntarily exit without sacrificing further medical treatment; safety issues; severe violation of the designed program by the patient; recruitment problems, such as patient not meeting the inclusion criteria; and loss to follow-up.

### Termination program

The reasons and any possible adverse events shall be inquired from patients who decide to terminate the study, and the observation and evaluation shall be performed by the researcher *per se* if possible. Serious or unexpected adverse events shall be closely followed up. The researcher should try as much as possible to acquire patient information from the concerned individual to complete the final evaluation, and the efforts should be recorded as well. The evaluation and observation report as well as the causes of exit described by patients should be recorded as primary data. The major causes of exit will be kept in CRF, and the subsequent procedures, such as follow-up examination after discharge, should be completed as much as possible.

## Discussion

The development course of the surgical procedure for NSCLC has been quite similar to that of breast cancer, going through a revolution from extensive resection to better organ preservation. The history of lung cancer operation began with pneumonectomy, which was first performed by Gramham in 1933 [35] and then regarded as a standard procedure. Later, in the 1960s, it was demonstrated that lobectomy could yield equivalent radical therapeutic effects while achieving better preservation in lung function compared with pneumonectomy. Thereafter, anatomical lobectomy in combination with lymph node dissection or sampling has become the standard surgical approach for NSCLC [36].

Thanks to the application of CT scan, an increasing number of asymptomatic NSCLC cases, even those with an ‘inert’ biological behavior, are identified at an early stage. Therefore, sublobar resection, including wedged resection and segmentectomy, has attracted academic attention. A comparative study on lobectomy and sublobar for stage I NSCLC, conducted by the LCSG, is the only randomized controlled trial so far and was completed and published in 1995. Their findings suggested that local recurrence is increased threefold in sublobar resection compared with lobectomy, and long-term survival rate was also slightly lower in sublobar resection, although there was no statistically significant difference [10]. The study confirmed the therapeutic effect of lobectomy and refuted sublobar resection; however, this conclusion remains highly controversial because the enrolled tumor size was considered too large for the sublobar resection group [37].

On the other hand, as the LCSG study implied that sublobar resection might not be suitable for all NSCLC patients with tumor size of less than 3 cm, sublobar resection might be more suitable for NSCLC with smaller size or for patients with high-risk factors as a ‘compromised’ option. That is how the two mainstream viewpoints for sublobar resection emerged and influenced the subsequent studies. Accumulating retrospective studies have compared sublobar resection and lobectomy in terms of long-term therapeutic effects [10, 24–26, 38–61]. Their results suggested that sublobar resection could achieve similar long-term outcomes as standard lobectomy for patients with early-stage NSCLC (diameter of not more than 2 cm) [40, 44, 45, 50–53, 55, 59, 61]; on the other hand, lobectomy may not convey more beneficial effects for the elderly with NSCLC of not more than 3 cm [24, 25, 39, 43, 47, 48, 57, 62]. However, prospective randomized controlled trials to support these assumptions are scarce.

Two randomized controlled trials assessing sublobar resection for less advanced NSCLC have been launched: CALGB140503 [63] (US) and JCOG0802 [37] (Japan). Both of them aim to verify the radical therapeutic effects of sublobar resection for NSCLC with a diameter of not more than 2 cm. Given that ‘radical effect’ is the focus of both trials, they may not elicit high-quality supportive evidence on the priority of sublobar resection for elderly patients, in whom sublobar resection may match radicalness with compromise.

Radical surgery may not convey sufficient benefits given the limited life expectancy of elderly patients. In addition to concurrent diseases and declined organ function, radical surgery is very likely to increase the operation risk. Therefore, a reasonably balanced strategy considering radical effect, operation risk, and life quality is needed for elderly patients with NSCLC. However, elderly patients with lung cancer are under-represented by far, and the present study is the first prospective randomized controlled trial on elderly patients with NSCLC at an early stage.

Given the complexity of life expectancy and operation risks, CGA is adopted in the present study to differentiate from trials evaluating young patients, in order to facilitate stratified randomization and analysis.

There are two parts of CGAs, the 4-year survival scale of Lee et al. [64] and CGA, which evaluate the benefit of operation by life expectancy and the intervention by operation risk, respectively.

First of all, non-tumor-caused death is common in elderly patients with early NSCLC, which indicates the need for stratification. However, such relevant studies to serve as references to the present study are rare. The 4-year mortality in the American cohort was analyzed by Lee et al., who also established the 4-year survival scale [64], which is consistent with a review published in *JAMA* (2012) [30]. Therefore, the 4-year survival scale established by Lee et al. was adopted in the present study as the standard of stratification. Patients will be classified into four groups: low risk (score of between 0 to 5 and 4-year mortality of 4 %), moderate risk (score of between 6 and 9 and 4-year mortality of 15 %), high risk (score of between 10 and 13 and 4-year mortality of 42 %), and extremely high risk (score of more than 14 and 4-year mortality of 64 %).

In the present study, the elderly patients fulfilling the operation criteria will undergo CGA, which is a multidisciplinary comprehensive assessment for the elderly, including assessments of organ function, concurrent diseases, cognitive function, psychological state, social and economic issues, syndromes, medications, and nutritional status. Recent studies showed that CGA could predict death and complication risks [65]. Although the CGA procedure is complex, its feasibility has been proven by the CALGB 360401 clinical trial [66].

Based on CGA, the PACE scale proposed by Audisio et al. in 2003 was particularly designed for elderly patients with tumor undergoing operation [67]. The PACE scale is a reinforced CGA and includes CGA, brief fatigue inventory, performance status, instrumental activities of daily living, activities of daily living, and the ASA scale. The PACE scale has a predictive value in elderly patients undergoing surgical operation, as demonstrated by multiple-center clinical validation [68].

Given the complexity of the CGA procedure, our lack of experience in geriatric assessments, and lack of practice on standardized and large-scale trials, the participants of the present study will be screened first by the VES-13 scale before CGA. VES-13 is a self-checklist, including age and 12 items of self-assessment concerning health and organ function. Compared with conventional CGA, VES-13 is more convenient and is capable of predicting the vulnerability of elderly patients and can be used as a pre-screening tool for CGA [31, 32]. In the present study, patients with a score of at least 3 will be considered high-risk subjects and followed up with PACE evaluation.

The present study is so far the only prospective, multicenter, randomized controlled trial comparing sublobar resection and lobectomy for elderly patients that aims to identify the most appropriate surgical approach for NSCLC at an early stage, to find the balance between radical effect and operation risk, and to assess survival period and life quality.

If the proposed study hypothesis is verified in the current trial, sublobar resection will be demonstrated to achieve equivalent therapeutic outcomes compared with lobectomy for elderly patients (≥70 years) with NSCLC at an early stage, with respect to DFS time, hospital stay, intubation time, peri-operative complication, death rate, overall survival rate, recurrence and metastasis rates within post-operative 3 and 5 years, and pulmonary function 6, 12, and 36 months after operation, respectively.

### Current status

The trial has received ethics approval, and enrollment in the trial has begun but has not yet reached full enrollment.

## Abbreviations

ASA: American Society of Anesthesiologists
CGA: Comprehensive geriatric assessment
CRF: Case report form
CT: Computed tomography
DFS: Disease-free survival
LCSG: Lung Cancer Study Group
NSCLC: Non-small cell lung cancer
PACE: Pre-operative assessment of cancer in the elderly
QoL: Quality of life
SEER: Surveillance, Epidemiology, and End Results
VES-13: Vulnerable Elders Survey
